# Multiomics-empowered Deep Phenotyping of Ulcerative Colitis Identifies Biomarker Signatures Reporting Functional Remission States

**DOI:** 10.1093/ecco-jcc/jjad052

**Published:** 2023-03-24

**Authors:** Lukas Janker, Dina Schuster, Patricia Bortel, Gerhard Hagn, Samuel M Meier-Menches, Thomas Mohr, Johanna C Mader, Astrid Slany, Andrea Bileck, Julia Brunmair, Christian Madl, Lukas Unger, Barbara Hennlich, Barbara Weitmayr, Giorgia Del Favero, Dietmar Pils, Tobias Pukrop, Nikolaus Pfisterer, Thomas Feichtenschlager, Christopher Gerner

**Affiliations:** Department of Analytical Chemistry, Faculty of Chemistry, University of Vienna, Vienna, Austria; Department of Analytical Chemistry, Faculty of Chemistry, University of Vienna, Vienna, Austria; Department of Analytical Chemistry, Faculty of Chemistry, University of Vienna, Vienna, Austria; Department of Analytical Chemistry, Faculty of Chemistry, University of Vienna, Vienna, Austria; Department of Analytical Chemistry, Faculty of Chemistry, University of Vienna, Vienna, Austria; Joint Metabolome Facility, University of Vienna, Vienna, Austria; Department of Analytical Chemistry, Faculty of Chemistry, University of Vienna, Vienna, Austria; Joint Metabolome Facility, University of Vienna, Vienna, Austria; Department of Analytical Chemistry, Faculty of Chemistry, University of Vienna, Vienna, Austria; Department of Analytical Chemistry, Faculty of Chemistry, University of Vienna, Vienna, Austria; Department of Analytical Chemistry, Faculty of Chemistry, University of Vienna, Vienna, Austria; Joint Metabolome Facility, University of Vienna, Vienna, Austria; Department of Analytical Chemistry, Faculty of Chemistry, University of Vienna, Vienna, Austria; Institute of Pathology and Microbiology, Krankenanstalt Rudolfstiftung, Vienna, Austria; Division of General Surgery, Department of Surgery, Medical University of Vienna, Vienna, Austria; Institute of Pathology and Microbiology, Krankenanstalt Rudolfstiftung, Vienna, Austria; Institute of Pathology and Microbiology, Krankenanstalt Rudolfstiftung, Vienna, Austria; Core Facility Multimodal Imaging, Faculty of Chemistry, University of Vienna, Vienna, Austria; Department of Obstetrics and Gynaecology, Medical University of Vienna, Vienna, Austria; Department of Internal Medicine III, Hematology and Oncology, University Hospital Regensburg, Regensburg, Germany; Institute of Pathology and Microbiology, Krankenanstalt Rudolfstiftung, Vienna, Austria; Institute of Pathology and Microbiology, Krankenanstalt Rudolfstiftung, Vienna, Austria; Department of Analytical Chemistry, Faculty of Chemistry, University of Vienna, Vienna, Austria; Joint Metabolome Facility, University of Vienna, Vienna, Austria

**Keywords:** Multi-omics, tissue proteomics, oxylipins, metabolomics, blood plasma

## Abstract

**Introduction:**

Ulcerative colitis [UC] is a chronic disease with rising incidence and unclear aetiology. Deep molecular phenotyping by multiomics analyses may provide novel insights into disease processes and characteristic features of remission states.

**Methods:**

UC pathomechanisms were assessed by proteome profiling of human tissue specimens, obtained from five distinct colon locations for each of the 12 patients included in the study. Systemic disease-associated alterations were evaluated thanks to a cross-sectional setting of mass spectrometry-based multiomics analyses comprising proteins, metabolites, and eicosanoids of plasma obtained from UC patients during acute episodes and upon remission, in comparison with healthy controls.

**Results:**

Tissue proteome profiling indicated colitis-associated activation of neutrophils, macrophages, B and T cells, fibroblasts, endothelial cells and platelets, and hypoxic stress, and suggested a general downregulation of mitochondrial proteins accompanying the establishment of apparent wound healing-promoting activities including scar formation. Whereas pro-inflammatory proteins were apparently upregulated by immune cells, the colitis-associated epithelial cells, fibroblasts, endothelial cells, and platelets seemed to predominantly contribute anti-inflammatory and wound healing-promoting proteins. Blood plasma proteomics indicated chronic inflammation and platelet activation, whereas plasma metabolomics identified disease-associated deregulations of gut and gut microbiome-derived metabolites. Upon remission several, but not all, molecular candidate biomarker levels recovered back to normal.

**Conclusion:**

The findings may indicate that microvascular damage and platelet deregulation hardly resolve upon remission, but apparently persist as disease-associated molecular signatures. This study presents local and systemic molecular alterations integrated in a model for UC pathomechanisms, potentially supporting the assessment of disease and remission states in UC patients.

## 1. Introduction

Ulcerative colitis [UC], an inflammatory bowel disease [IBD], is characterised by ascending inflammation of the large intestine, with intermittent cycles of active inflammation and asymptomatic periods.^1^ With an early disease onset at around 30 years of age and increasing incidence rates, ie, 24.3/100 000 in Europe and 19.2/100 000 in North America, UC is becoming an increasing and severe health risk for millions of people.^2,3^ In relation to the chronic gut inflammation, UC patients suffer from an increased risk of colorectal cancer as well as thromboembolic complications.^4-6^ The chronicity of UC is accompanied by lifelong non-curative treatment of symptoms, which is challenging. Patients are treated based on symptom subsets and severity in an iterative scheme^1,7-10^ that has not fundamentally changed for the past two decades.^5,11,12^ The Ulcerative Colitis Disease Activity Index [UCDAI] and MAYO score rely on multiple clinical observations, invasive and non-invasive in nature, as well as on the compliance of patients regarding subjective measures in the form of patient-reported outcomes, called PRO reports.^13,14^

A vast effort was put into the elucidation of disease-driving genetic factors, including genome-wide association studies, but to the best of our knowledge, no definitive causal links can be established yet.^15^ MHC locus HLA Class II alleles, as well as the multidrug resistance gene *MDR1*, have been implicated as possible genetic susceptibility factors.^16-18^ In contrast to studies of UC, studies focusing on the genetic landscape of Crohn’s disease [CD] found evidence of causal effects of single nucleotide polymorphisms, with biological plausibility and dose-response effects independently verified by multiple studies.^19-22^

The above-mentioned points strengthen the theory of possible involvement of environmental exposures and other post-genomic related influencing factors, such as the general composition and functional state of the proteome, metabolome, and microbiome. Even for malignant diseases where genetic instability determines disease progression, post-genomic analysis tools can provide deep insights into molecular mechanisms of progression and escalation.^23^ Recent studies^24,25^ investigated potential pathophysiological mechanisms of UC, showing for example the influence of the microbiome and its compositional alterations before and after diagnosis with UC,^26^ the activation of the innate immune system monitored on a proteome-wide scale,^27^ or general compositional changes in the metabolome^28,29^ or microbiome-dependent co-metabolome.^30,31^ Although these findings are leading UC-related research into a more molecularly focused direction, there are still pathophysiological processes that remain to be unveiled. Studies focusing on *post hoc* analysis of serological markers for the discrimination of clinically relevant cohorts showed the limitations of established marker molecules, and demonstrated the need for comprehensive biomarker discovery and combination of data on multiple omics levels.^32,33^ The potential power of multiomics analyses to contribute to these challenges was already recognised, resulting in several studies successfully applying genome-wide association studies or integrating clinical phenotypes with environmental exposures relevant for UC.^34,35^

In the present study, we thus aimed to investigate pathophysiological aberrations which can be detected *in situ*. We carried out an in-depth analysis of intra-individual colonic proteome alterations during active UC in a spatially resolved manner. The parallel analysis of plasma samples, enabling the identification of systemic disease-associated alterations, was performed by the combination of an extensive panel of biomolecules ranging from amino acids to lipids, including eicosanoids and plasma proteins, as applied by us for other chronic diseases such as long COVID syndrome.^36^ An additional case-control study with UC patients in remission was performed in order to investigate remission-associated normalisation of biomarker profiles. Thus, we demonstrate that some colitis-associated biomarkers are still deregulated at a time when no more symptoms are reported and more likely indicate possible determinants for chronicity. The present multiomics analyses provide a deep molecular phenotyping of UC disease manifestation, pointing at novel therapeutic targets and presenting biomarker candidates that may be validated in prospective clinical trials.

## 2. Materials and methods

### 2.1. Study design and population

**Figure 1:**
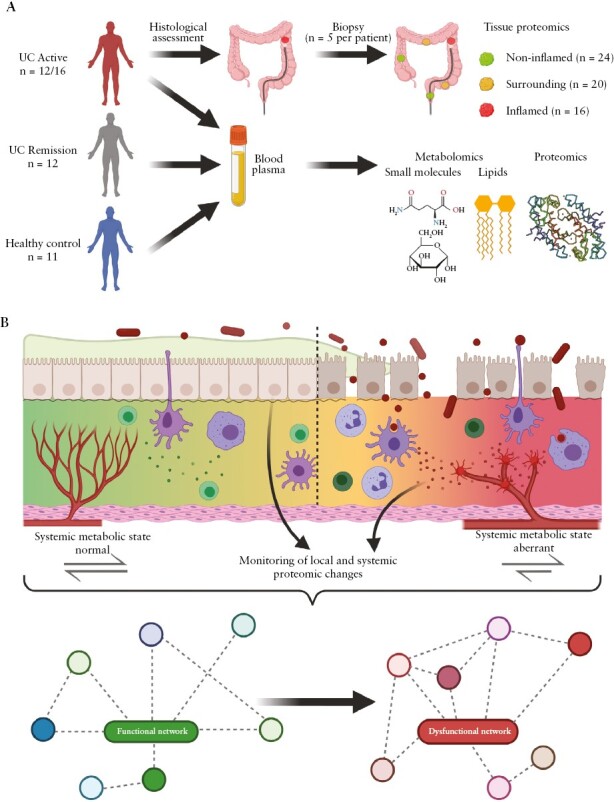
Study design supporting the discovery of biomarker profiles. [A] Patient cohort and general sampling strategy. Patients with active UC [top, *n* = 12 for complete tissue and plasma series, *n* = 4 only plasma available] providing tissue [*n* = 5 per patient] and plasma samples. Blood plasma samples from UC patients in remission [*n* = 12], as well as healthy controls [*n* = 11], were collected. Based on histological classification, tissue samples were categorised into non-inflamed [*n* = 24], surrounding [*n* = 20], and inflamed [*n* = 16] tissue. [B] Schematic overview regarding pathophysiological processes taking place in different compartments. Blood microvessels supply the mesenchymal interstitium separated from the mucus-forming parenchyma via a basal membrane. Disease progression [left to right] associated with microvascular damage, invasion of immune cells, breakdown of the basal membrane, loss of epithelial cells, and increased access of inflammation-inducing bacteria are depicted, together with the presently applied complementary analysis strategies. UC, ulcerative colitis.

The present case-control study includes patients from the age of 18 and above, diagnosed with ulcerative colitis [UC] in either active or remission state, as well as healthy control patients as described in Figure 1A. Patient information regarding clinical data from active and remission patients, as well as age distribution in regard to healthy controls, can be found in Supplementary Table S1 and Supplementary Figure S1. Inclusion criteria for active UC patients were diagnosis of UC with acute symptoms and a MAYO score of 3 or more. Exclusion criteria for active UC patients included pancolitis, remission status according to MAYO score, infections, and colon resection or colectomy, as well as colitis indeterminata. Inclusion criteria for UC patients in remission were diagnosis of UC with a recent history of acute disease symptoms but a current, complete resolution of symptoms, no rectal bleeding, normal stool frequency, and a physician’s rating of disease activity as normal. Exclusion criteria for remission UC patients included infections and colitis indeterminata, as well as colon resection or colectomy. Patients with active UC underwent a routine colonoscopy for the assessment of the local inflammatory status including faecal calprotectin and assignment of MAYO score.^13^ Colon biopsies were classified by histological assessment into three categories, ranging from non-inflamed to surrounding/mildly inflamed to inflamed. Classification was based on histological findings of inflammatory features [Supplementary Figure S2. For the monitoring of systemic events happening during the pathogenesis of UC, blood plasma was collected from each patient cohort.

### 2.2. Ethical statement

The study was approved by the Ethics Commission of the city of Vienna with votum EK 18-193-0918. All patients gave informed consent.

### 2.3. Sample collection and processing

During routine colonoscopy, biopsies of rectum, colon sigmoideum, colon descendens, colon transversum, and colon ascendens were split into two parts. The biopsy part designated for proteomic analysis was washed thoroughly with Ringer’s solution, placed in a cryo-tube and stored at -80°C. Blood plasma was collected by centrifugation of full blood mixed with EDTA at 1500 *g* for 10 min and subsequently stored at -80°C.

### 2.4. Plasma and tissue proteomics

Lysis buffer [8 M urea, 50 mM TEAB, 5% SDS] was added to EDTA plasma at a ratio of 1:10 and to tissue samples [100 µL] which were lysed via ultrasonic rod. Protein concentrations were determined via BCA-assay and 20 µg of protein was used for further processing. For protein digestion, a ProtiFi S-trap^TM^ protocol was employed as described previously.^37^ In short, proteins were reduced and alkylated using DTT and IAA, respectively, followed by addition of trapping buffer. Samples were loaded onto S-trap^TM^ cartridges and digested with Trypsin/Lys-C for 2h at 37°C. Supernatants containing the peptides were eluted, dried, reconstituted in 5 µL formic acid containing 10 fmol of four synthetic standard peptides and diluted with 40 µL mobile phase A. LC-MS/MS measurements were performed on a timsTOF Pro mass spectrometer [Bruker Daltonics] hyphenated with a Dionex UltiMate^TM^ 3000 RSLCnano system [Thermo Scientific]. Parameters for analyses were an adapted version of a previously published method^37^: 0.5 µL and 5 µL of plasma and tissue proteomic samples, respectively, were loaded onto an Acclaim^TM^ PepMap^TM^ C18 100 pre-column [Thermo Fisher Scientific] at a flow rate of 10 µL/min using mobile phase A, and eluted onto an Aurora Series emitter column [Ionopticks] applying a flow rate of 300 nL/min. Separation was achieved by applying a gradient of 8% to 40% mobile phase B over 55 min and 90 min for plasma and tissue samples, respectively. Data analysis was performed using MaxQuant [version 1.6.17.0] employing the Andromeda search engine for protein identification against the UniProt database [12/2019, 20 380 entries].^38^ Search parameters were set as previously described.^37^ Fixed modifications included carbamidomethylation of cysteine and methionine oxidation, N-terminal protein acetylation was set as variable modification. For the data evaluation in Perseus, data were grouped and filtered according to missing values. A total of five or three valid values had to be present in at least one cohort in tissue or plasma samples, respectively, to be considered a valid identification. Data were log2-transformed and imputation was performed according normal distribution with a down-shift of 1.8 sigma and a width of 0.3 sigma. For the group-wise comparison, two-sided t tests were performed.

### 2.5. Eicosanoid analysis

EDTA plasma [0.4 mL; freshly thawed on ice] was added to 1.6 mL cold ethanol [EtOH, abs. 99%, -20°C; AustroAlco] containing 100 nM of each internal standard [12S-HETE-d8, 15S-HETE-d8, 5-Oxo-ETE-d7, 11.12-DiHETrE-d11, PGE-d4, 20-HETE-d6; Cayman Europe] and kept overnight at -20°C to allow for protein precipitation. The suspension was centrifuged for 30 min at 4°C [4536 g, 9 DCC, 7 ACC] and the supernatant was transferred to a new 15-ml FalconTM tube. Afterwards, ethanol was evaporated via vacuum centrifugation at 37°C until the original sample volume was restored. Samples were loaded on preconditioned StrataX solid phase extraction [SPE] columns [30 mg/mL-1; Phenomenex] using Pasteur pipettes and washed with 5 mL MS grade water, and elution of eicosanoids was achieved with 500 µL ice-cold methanol [MeOH abs.; VWR International] containing 2% formic acid [FA; Sigma-Aldrich]. Methanol was evaporated under a gentle N2 stream at room temperature and dried samples were reconstituted in 150 µL reconstitution buffer [H2O/ACN/MeOH + 0.2%FA - 65:31.5:3.5], including another set of 10-100 nM internal standards [5S-HETE-d8, 14.15-DiHETrE-d11, 8-iso-PGF2a-d4; Cayman Europe, Tallinn, Estonia].

Eicosanoids were analysed with a Thermo ScientificTM VanquishTM [UHPLC] system coupled to a Q ExactiveTM HF Quadrupole-OrbitrapTM mass spectrometer [Thermo Fisher Scientific, Austria], equipped with an HESI source for negative ionisation. In short, eicosanoids were separated on a Kinetex® C18-column [2.6 μm C18 100 Å, LC Column 150 x 2.1 mm; Phenomenex®] at a flow rate of 200 µL/min-1. Per injection, 20 µL of the sample was loaded and all samples were analysed in two technical replicates. The 20-min UHPLC method included a gradient flow profile [mobile phase A: H_2_O + 0.2% FA, mobile phase B: ACN:MeOH [90:10] + 0.2% FA] starting at 35% B increasing to 90% B [1–10 min], further going up to 99% B in 0.5 min and held for 5 min. Afterwards, solvent B was decreased to a level of 35% in 0.5 min and the column was equilibrated for 4 min. The column oven temperature was set to 40°C. Mass spectrometric resolution on the MS1 level was set to 60 000 [at m/z = 200] with a scan range from 250 to 700 m/z. The two most abundant precursor ions were selected for fragmentation [HCD 24 normalised collision energy], preferentially from an inclusion list containing 31 m/z values specific for eicosanoids and their precursor molecules. The resulting fragments were analysed on the MS2 level at a resolution of 15 000 [at m/z = 200]. Operating in negative ionisation mode, a spray voltage of 2.2 kV and a capillary temperature of 253°C were applied. Sheath gas was set to 46 and the auxiliary gas to 10 arbitrary units. Raw files generated by the Q ExactiveTM HF Quadrupole-OrbitrapTM mass spectrometer were checked manually using Thermo XcaliburTM 4.1.31.9 [Qual browser]. Spectra were compared with reference spectra from the Lipid Maps depository library from July 2018^.40^ Peaks were integrated using the TraceFinderTM software package [version 4.1 - Thermo Scientific].

### 2.6. Targeted metabolomics experiments

EDTA plasma samples [10 µL] of patients were analysed by a targeted metabolomic assay. Targeted metabolomics experiments were conducted using the MxP® Quant 500 Kit [Biocrates Life Sciences AG], which enables the detection and [semi]quantification of up to 631 analytes, including 40 acylcarnithines, 1 alkaloid, 1 amine oxide, 50 amino acid-related metabolite, 15 bile acids, 9 biogenic amines, 7 carboxylic acids, 28 ceramides, 22 cholesteryl esters, 1 cresol, 44 diacylglycerols, 8 dihydroceramides, 12 fatty acids, 90 glycerophospholipids, 34 glycosylceramides, 4 hormones, 4 indole derivatives, 2 nucleobase-related metabolites, 15 sphingolipids, 242 triacylglycerols, the sum of hexoses, and 1 vitamin/cofactor. A total of 494 metabolites showed signal intensities within the quantification window and were further evaluated. Measurements were carried out using LC-MS and flow injection [FIA]-MS analyses on a Sciex 6500+ series mass spectrometer coupled to an ExionLC AD chromatography system [AB Sciex], using the Analyst 1.7.1 software with hotfix 1 [AB SCIEX]. All required standards, quality controls, and eluents were included in the kit, as well as the chromatographic column for the LC-MS/MS analysis part. Phenyl isothiocyanate [Sigma-Aldrich] was purchased separately and was used for derivatisation of amino acids and biogenic amines according to the kit manual. Preparation of the measurement worklist as well as data validation and evaluation were performed with the software supplied with the kit [MetIDQ-Oxygen-DB110-3005, Biocrates Life Sciences].

### 2.7. Statistical data analysis

Proteomics data were analysed with MaxQuant [version 1.6.17.0].^38^ Eicosanoid spectra were compared with reference spectra from the Lipid Maps depository library and subsequently integrated using the TraceFinderTM software package [version 4.1].^40^ Targeted metabolomics data validation and evaluation were performed with the software supplied with the MxP® Quant 500 Kit [MetIDQ-Oxygen-DB110-3005]. For the analysis of causal networks between inflamed and non-inflamed tissue, the software CausalPath with standard parameter settings and pre-processed label free quantification (LFQ) intensity values was used.^41^ Additional statistical analysis was performed with Perseus [version 1.6.14.0], Microsoft Excel, and GraphPad Prism [version 6.07].

Omics data were integrated in R using N-integration discriminant analysis with DIABLO [Data Integration Analysis for Biomarker discovery, R-package mixOmics 6.18.0,^42]^ and an own-developed method using Gaussian Graphs Models Selection [R-package GGMselect 0.1-12.4, CIT2] for analytes reduction via sub-network generation and principal component representation. Subsequently, partial correlations of significantly deregulated single analytes with analyte sub-network representations were observed.^43^ All eicosanoid values that were below the detection limit were imputed with the respective minimal value divided by the square root of two, and all values were sub sequentially log_2_-transformed.

A more fine-grained analysis following the methods described in Svoboda *et al.*,^44^ Bekos *et al.*,^43^ and Muqaku *et al.*,^45^ with the following steps were performed. For all selection steps in the following analysis, a false-discovery rate [FDR] cutoff of 5% was chosen.

1] For all three analyte types, single analytes significantly with the differences between i] healthy controls and acute diseased patients, ii] healthy controls and patients in remission, and iii] patients in remission and acute diseased ones [*ie,* the so called contrasts], were determined using linear modelling and an empirical Bayesian approach to moderate the standard errors across analytes, *ie,*, shrink towards a common value, as implemented in the R-package limma.^46^ Information of age and sex was included in the models and therefore results were corrected for these two possible confounders.

2] Using graphical Gaussian modelling [GGM] implemented in R-package GGMselect^47^ for each analyte type, sub-networks were identified and tested if significantly associated with the contrasts under analysis and–if yes–were used for subsequent integration over all analyte types. This step was done to reduce the number of analytes for a more clearly represented integration and easier interpretation. The principle behind GGM is to use partial correlations as a measure of independence of any two analytes, which allows for distinguishing direct from indirect interactions. The tuning parameter K for the penalty function was varied between 1 and 6 in 0.5 step, and the function *selectFast* (*family = c[‘C01’, ‘LA’,])* was used for optimising the model employing the C01 and the lasso and [LA]-algorithm [https://cran.r-project.org/web/packages/GGMselect/vignettes/Notice.pdf]. At each K, the resulting analyte sub-networks were used for gene set analyses [GSA, using raw *p*-values and regulation direction from the single analyte analyses with function *runGSA*, implemented in R-package piano] to determine the significant associations with all contrasts. Finally, the K-value was chosen which yielded the largest number of contrast-specific, significantly associated sub-networks. All of these significantly associated sub-networks were summarised by the first [PC1] or first two principal components [PC1 and PC2; if the proportion of the explained variance of the first component was below 75%].

3] For each contrast, all principal components for all significant sub-networks and all remaining significant single analytes over all omics types were collected together with the information on age and sex, and partial correlations were calculated between all of them [R-package ppcor v1.1].^48^

4] Finally, a network was plotted for every contrast showing all significant single analytes and sub-networks (represented by the first [two] principal components) and their partial correlations with each other [cutoff |R| >0.6]. Colour of nodes [single analytes] and pie pieces of nodes [members of each sub-network] represent the log_2_ fold-change between the two compared groups of each contrast, and the colour of the edges represents the R-value of the correlations between the nodes.

### 2.8. Evaluation of publicly available transcriptomics data

Data [E-MTAB-7860 and E-MTAB-9731] were accessed via ArrayExpress.^49,50^ Fastq files where downloaded via European Nucleotide Archive and aligned to GRCh38 using kallisto [https://doi.org/10.1038/nbt.3519].^51^ Abundance ­estimates where imported into R using the package txi import. Differential gene expression analysis was done using DESeq2 with the model Y ~ 0 + HEALING + STUDY, with HEALING being the disease status [normal, not healed, and healed] and STUDY being the dataset [10.12688/f1000research.7563.1, 10.1186/s13059-014-0550-8]; *p*-values were adjusted for multiple testing according to Benjamini and Hochberg.^52^

## 3. Results

### 3.1. Rationale and presentation of the study design

After informed consent, five colon biopsy samples from different locations were obtained from each of the 12 patients experiencing active ulcerative colitis [clinical parameters are provided in Supplementary Table S1]. The tissue samples were categorised via histological analysis into ‘inflamed’, ‘surrounding’, and ‘non-inflamed’ [Figure 1A; Supplementary Figure S1]. Blood plasma was collected from a total of 16 UC patients in acute episode, including the 12 patients who underwent biopsy, as well as another 12 UC patients after successful remission [free of symptoms, all relevant clinical parameters normalised as listed in Table 1 and documented in Supplementary Figure S2, Supplementary Table S1, S2]. Blood plasma from 11 healthy donors served as control. Each plasma sample was analysed with regard to proteins, metabolites, and eicosanoids, using three different mass spectrometry-based analysis methods [Figure 1A]. Tissue proteome profiling was conducted to provide insight into disease-associated pathomechanisms, and plasma samples from the same patients were analysed to investigate whether systemic alterations might be directly related to local pathomechanisms [Figure 1B].

**Table 1.** Patient cohort description

	Active	Remission
Age [years]	47 ± 16	44 ± 13
Sex [m/f]	3/9	5/5
Duration of illness [years]	15 ± 9	18 ± 11
Mayo score [mdcalc.com]	**5.9 ± 2.7**	0.3 ± 0.7*
Leukocytes [g/L]	**9.7 ± 1.8**	6.6 ± 2*
Thrombocytes [g/L]	**352.8 ± 65.6**	247.8 ± 61.7*
Erythrocytes [T/L]	4.7 ± 0.6	4.9 ± 0.4
CRP [mg/L]	9.1 ± 17.5	3.8 ± 4.7
Albumin [g/L]	42.5 ± 4.8	43.3 ± 5.7
Calprotectin/ST [µg/g]	**811.5 ± 812.4**	10.4 ± 6.2*

* *p*-value ≤0.05 according to Student’s t-test, significant differences between active UC and remission state are indicated in bold. Numerous other clinical parameters documented in Supplementary Table S1 were not significantly different between the two groups. ST, measurement with stool samples.

### 3.2. Tissue proteomics reveals molecular patterns associated with local UC pathomechanisms

**Figure 2.**
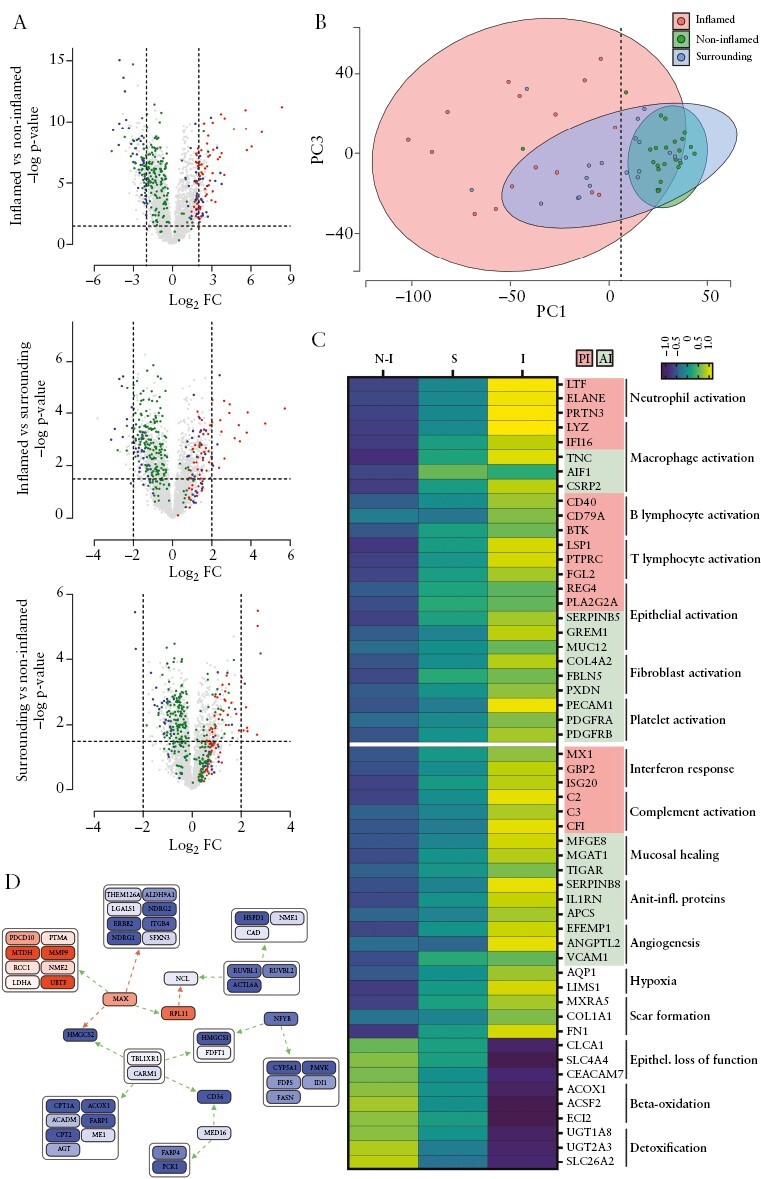
Tissue proteomics identifies pathomechanisms characteristic for UC. [A] Volcano plots illustrating comparisons of differentially abundant proteins in tissue samples according to histological graded categories. Proteins highlighted correspond to neutrophil granulocytes/macrophages [red], epithelial/fibroblast cells [blue], and mitochondria [green]. VIM and KRT18 are highlighted as an orange triangle or square, respectively. Axes were scaled according to extend of regulation, with anchor points as dotted lines at -log *p*-value of 1.5 [horizontal] and log2-fold change of -2 and 2 [vertical]. [B] Principal component [PC] analysis of tissue proteomics dataset, each point representing a single colon biopsy. PC1 is plotted against PC3, allowing a complete separation of non-inflamed sections from inflamed sections [dotted line], with surrounding sections in between. [C] Heatmap of selected proteins of interest involved in pathophysiological processes. Colour coding of gene names with red and green relates to pro-inflammatory [PI] and anti-inflammatory [AI] properties of the given protein, respectively. Label free quantification (LFQ) intensity values were z-score normalised and averaged for inflammation-related grouping. [D] Causal-path analysis results of comparison from inflamed vs. non-inflamed tissue. Protein modules, coloured from blue [lower expression] to red [higher expression], linked via direct positive [green] or negative [red] connectors form nodes of uniformly regulated proteins which are combined in hubs. For the calculation of significant causal connected regulation events, standard parameters were employed. UC, ulcerative colitis.

The analysis of a total of 60 tissue biopsy samples identified 4579 proteins [at least two peptides per protein, FDR <0.01 at protein and peptide level, at least five independent identifications in at least one tissue category per protein] and various protein regulatory events distinguishing the three predefined tissue categories [Figure 2A]. Principal component analysis [PCA] separated ‘inflamed’ versus ‘non-inflamed’, and the surrounding tissue samples were found dispersed across the other two groups [Figure 2B]. Due to cell invasion events typically associated with inflammation, the cell type composition differs when comparing the tissue categories. Thus, differentially regulated proteins [FDR <0.01] were attributed in a first step to cell types according to expression specificity. In a subsequent step, functional cell activation markers were considered as described previously,^53-55^ in addition to protein regulatory events pointing to characteristic pathomechanisms [Figure 2C; Supplementary data, part 4].

The inflammation-associated upregulation of VIM accompanied by a downregulation of KRT18 [Figure 2A; Supplementary Table S2] indicated a relative increase of mesenchymal cells at the cost of parenchymal cells. This interpretation was corroborated by histological assessments; furthermore, many neutrophil-, macrophage-, and fibroblast-specific proteins were identified among the most strongly upregulated proteins in inflamed tissue [Figure 2A, C]. Several epithelial proteins known to be induced upon inflammation, such as REG4, PLA2G2A, and GREM1, were also found upregulated. In contrast, epithelial proteins characteristic for colon functions such as mucus formation were downregulated, including CLCA1, SLC4A4, and CEACAM7. Actually, mitochondrial proteins were the protein group most consistently downregulated [Figure 2A, C].

The significant upregulation of characteristic marker proteins [Figure 2C; Supplementary Table S2 tissue marker proteins] in inflamed tissue samples demonstrated the activation of neutrophils [LTF, ELANE, and PRTN3], macrophages [LYZ, IFI16], B lymphocytes [CD40, CD79A, BTK] and T lymphocytes [LSP1, PTPRC, and FGL2]. These molecular signatures were further associated with an interferon response pattern [MX1, GBP2, ISG20] and local involvement of complement factors [C2, C3, CFI]. Importantly, local functional platelet activation was indicated by the upregulation of PECAM1, PDGFRA, and PDGFRB. The observed upregulation of FBLN1 and CD40 may represent potential promoters for such platelet activation, and indicate an onset of wound-healing processes already during acute UC. Activated platelets are known to release large amounts of TGF-β,^56^ potentially acting in an anti-inflammatory fashion and characteristic for systemic inflammatory response syndrome.^57^ Indeed, many characteristic TGF-β induced proteins derived from macrophages [eg, TNC, AIF1] and epithelial cells [eg, SERPINB5, GREM1, MUC12] were found significantly upregulated. The facilitation of myofibroblast formation by TGF-β, as described previously^58^ in inflamed colon tissue samples, was indicated by the increase of COL4A2, FBLN5, and PXDN. In addition, markers for mucosal healing [eg, MFGE8, MGAT1, TIGAR] and anti-inflammatory proteins such as SERPINB8, IL1RN, and APCS, were found upregulated.

Moreover, a proteome signature for hypoxia [CSRP2, AQP1, LIMS1] was evidenced. Hypoxia is capable of promoting angiogenic proteins, as presently detected in case of EFEMP1, ANGPTL2, VCAM1. Hypoxia may also promote scar formation,^59^ as evidenced by the characteristic scar constituents MXRA5, COL1A1, and FN1 [Figure 2C]. The downregulation of enzymes essential for beta-oxidation, such as ACOX1, ACSF2, and ECI2, in inflamed tissue samples indicates the involvement of mitochondria as mentioned above, potentially resulting in a loss of energy-demanding detoxification capacity. Indeed, the detoxification enzymes UGT1A8, UGT2A3, SLC6A2, and others were found significantly downregulated.

Investigation of the tissue proteomics data using causal path analysis [see Materials and Methods] indicated a hub function of the metabolic key transcription factor MAX [Figure 2D], which was previously linked to a hypoxia-induced metabolic switch promoting glycolysis above beta-oxidation.^60^

Two independent strategies were used to confirm the present findings. First, transcriptomics data from human colitis tissue samples publicly available via ArrayExpress were analysed. ^49,50^ As demonstrated in Supplementary Table S3, the mRNA corresponding to all but two of the 54 marker proteins reported and discussed above were detected by transcriptomics. Indeed, all genes corresponding to the upregulated proteins were also found upregulated in the gene sets, 19 of 45 in a significant fashion [adjusted *p*-values below 0.05]. Furthermore, all genes corresponding to the downregulated proteins were also found downregulated, here five out of nine in a significant fashion.

In a second approach, the present tissue proteome profiles were compared with a previously published colon tissue study comprising colon carcinoma and neighbouring normal colon tissue samples.^61^ Of the 54 marker proteins, 46 had been detected as well [Supplementary Table S4]. The epithelial activation markers REG and SERPINA5, the interferon response marker MX1, and the anti-inflammatory protein IL1RN were found significantly deregulated in tumour tissue, similar to the inflamed tissue. However, other UC-associated events, such as B and T lymphocyte, fibroblast, platelet, and complement activation, were not evidenced in tumour tissue samples, whereas epithelial loss of function and a loss of detoxification competence were apparently similar but did not reach significance thresholds.

### 3.3. Blood plasma alterations accompany acute UC episodes

**Figure 3:**
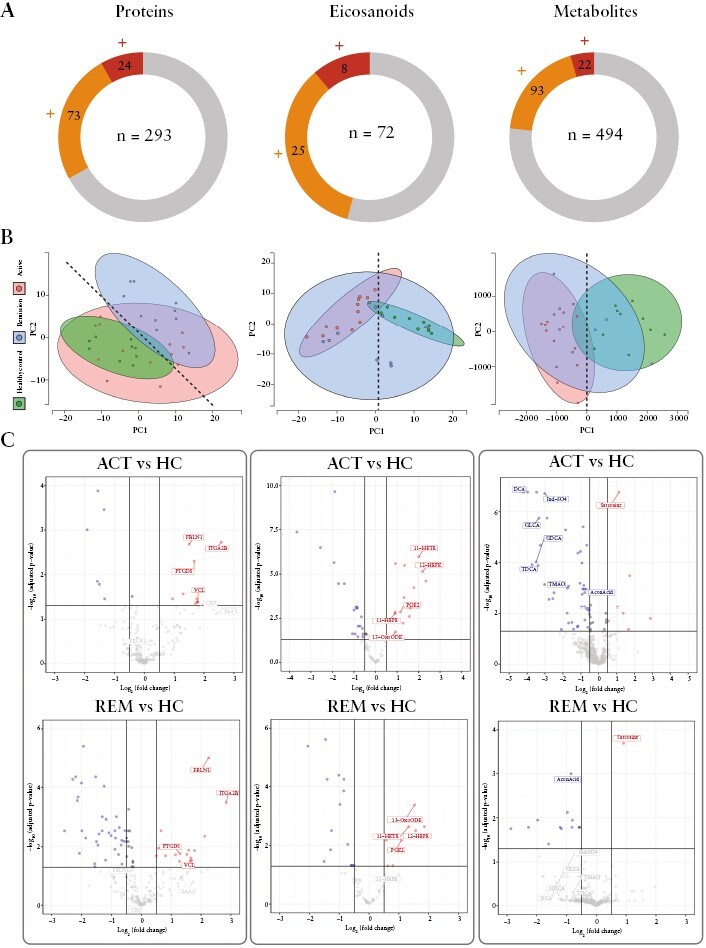
Multiomics analysis of blood plasma. [A] Pie charts indicating significant regulations of proteins, eicosanoids, and metabolites derived from plasma samples. Number of experimentally determined analytes per molecule class are depicted in the middle of the respective pie chart circle. Statistical testing was performed employing a two-sided t test [*p* = 0.05] for the comparison between 1] UC Active versus Healthy Control and 2] UC Remission versus Healthy control. Number of analytes only significantly regulated in one of those comparisons are depicted in orange [+], analytes significantly regulated in both comparisons are depicted in red [++]. [B] Principal component analysis [PCA] of illustrated datasets. Individual dots represent individual patients; 95% confidence intervals of patient cohorts are highlighted via respective colouring of areas in the plot. PCA of plasma proteins allows separation of Healthy Controls from UC Remission patients over both axes [dotted line], PCA of plasma metabolites as well as eicosanoids allowed separation of Healthy Controls from UC Active patients via PC1 [dotted lines]. [C] Volcano plots of comparative data analysis as indicated. Significant protein regulatory events [-log *p*-value = 1.5] between patient cohorts are highlighted in blue [downregulation] and red [upregulation]. Selected molecules are annotated. UC, ulcerative colitis.

Proteins, eicosanoids, and metabolites were analysed in blood plasma samples derived from healthy controls and patients suffering from an acute episode of UC, as well as UC patients after remission. Three different LC/MS-based analysis strategies were used, resulting in the comparative analysis of 293, 72, and 494 distinct molecules on the levels of proteins, eicosanoids, and metabolites, respectively [Figure 3A; Supplementary Table S2]. Unsupervised PCA of identified plasma proteins separated the remission group from the healthy group, whereas the PCA of metabolomics and eicosanoid analyses rather separated active disease patients from healthy controls [Figure 3B]. Each omics analysis delivered significant regulatory events as displayed in volcano plots [Figure 3C; and documented in Supplementary Table S2].

In a knowledge-based approach for data interpretation, molecules deregulated in plasma of UC patients were screened manually, searching for potential biomarkers related to pathomechanisms evidenced by tissue proteomics. Actually, the clinical markers C-reactive protein [CRP] and SAA1, indicative for acute inflammation, were found upregulated by proteomics in UC patients, but missed significance thresholds [Figure 3C]. The following molecules were discriminated based on significant alterations within one or two, out of two, group comparisons [active UC versus control and remission versus control, Figure 3A]. The upregulated proteins ITGA2B, FBLN1, and VCL are strongly expressed by platelets^62^ and demonstrate systemic involvement of platelets in UC. Remarkably, the platelet-inhibiting enzyme PTGDS, described to be upregulated upon injury,^63^ was also found strongly upregulated.

The eicosanoid analyses provided further independent support of a systemic platelet involvement in UC. Six out of 12 eicosanoids and fatty acids, presently identified to be significantly and more than 2-fold upregulated [Supplementary Table S2], were described to be released by activated platelets.^64^ Besides the pro-inflammatory PGE2, they comprised the anti-inflammatory molecules 13-OxoODE, 11-HEPE, 12-HEPE, 11-HETE, 12-HETE, and 10-HDoHE [Figure 3C]. The latter molecules were previously described to be also associated with a neutrophil eicosanoid class switch characteristic for alternatively polarised neutrophils [N2].^45^ Further, 13-OxoODE has been described as product of acetylated COX-2 resulting from aspirin consumption^65^ and is an endogenous ligand for PPARγ in human colonic epithelial cells.^66^ Thus, pro- and anti-inflammatory contributions from platelets and potentially neutrophils characterised the UC-associated plasma eicosanoid profile.

Notably, metabolomics analyses detected the downregulation of several amino acids including ornithine. Increased levels of propionylcarnitine [C3] and a loss of aconitic acid [AconAcid], an essential citric acid cycle component, may indicate mitochondrial stress. Increased plasma cystine levels are typically interpreted as oxidative stress.^67^ The downregulation of the primary bile acids including glycocholic acid, and the secondary bile acids deoxycholic acid [DCA], glycodeoxycholic acid [GDCA], and glycolithocholic acid [GLCA], demonstrates dysregulation of lipid homoeostasis. Also, metabolites characteristic for the gut microbiome, such as p-cresol sulphate, 3-indol propionic acid, trimethylamine N-oxide, and indoxyl sulphate [Ind-SO_4_], were found significantly decreased. Sarcosine, which has been described as oncometabolite due to its implications with hypoxia and mitochondrial stress,^68^ was actually found significantly upregulated.

Several metabolic alterations may be directly related to associated enzymes, as presently observed by tissue proteomics. Along with many other mitochondrial proteins, ACO2 was found downregulated in inflamed tissue regions, as well as cytoplasmic ACO1 [Figure 2A]. This may relate to the systemic downregulation of aconitic acid mentioned above. CYP27A1, another mitochondrial cytochrome P450 enzyme presently found downregulated in inflamed tissue, catalyses the first biosynthetic step of bile acid synthesis, and may thus contribute to the above-described downregulation of bile acids. LPCAT1, an enzyme found significantly upregulated in inflamed tissue samples, was described to be upregulated upon gut microbiome dysregulation.^69^ The main substrates consumed by LPCAT1, lysophosphatidylcholines [*n* = 14 distinct molecules] were found significantly downregulated in the plasma of colitis patients [Supplementary Table S2]. This may promote the formation of platelet-activating factors [PAFs],^70^ resulting in platelet activation and perpetuation of chronic inflammation by calcium mobilisation.

### 3.4. Modelling the multiomics data suggests unresolved disease processes upon remission

Remission from acute UC was diagnosed by physicians according to cardinal symptoms such as normal stool frequency and absence of rectal bleeding. The MAYO score and the blood parameters leukocyte and thrombocyte counts, as well as calprotectin detection in stool, were significantly downregulated in comparison with patients with acute UC [Table 1]. Only patients showing full remission were included. Remarkably, through comparison of control, remission, and active UC plasma samples, we identified several candidate biomarkers not recovering back to healthy control levels. Figure 3C demonstrates that proteome alterations observed in patients after remission, when compared with healthy controls, hardly differ from acute UC episode-associated alterations. This suggested that the systemic alteration of platelet functions persisted after clinical remission. Similarly, the platelet-associated eicosanoids PGE2, 11-HEPE, 12-HEPE, and 11-HETE hardly returned to normal levels. Also, the oncometabolite sarcosine and the mitochondrial metabolite aconitic acid remained deregulated after remission. On the other hand, the levels of bile acids DCA, GDCA, and GLCA and the gut microbiome-derived metabolites Ind-SO_4_, 3-IPA, and 3-IAA were found close to normal values upon remission, potentially indicating efficient recovery of gut functions such as lipid resorption and gut microbiome metabolism.

**Figure 4:**
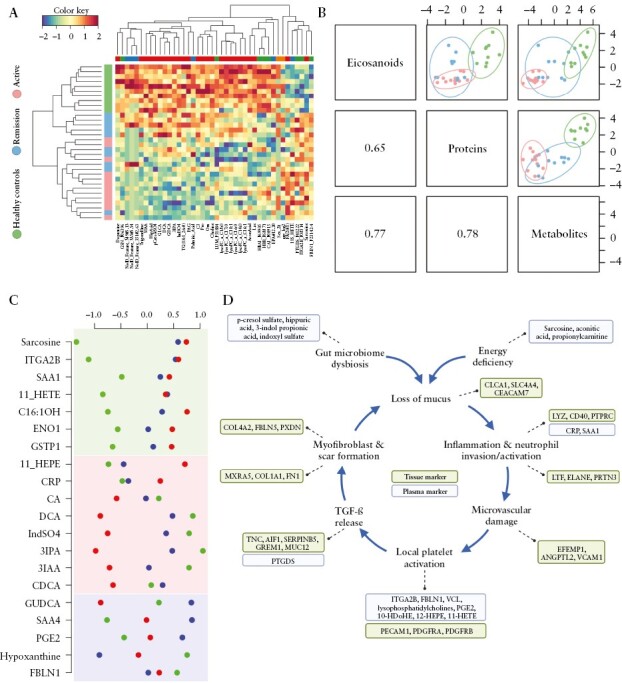
Data integration analysis and prognostic biomarker candidates. [A] Heatmap of plasma analytes [proteins, eicosanoids, and metabolites], selected by Data Integration Analysis for Biomarker discovery [DIABLO] implemented in the R-package mixOmics including information on age and sex. Eight proteins, six eicosanoids, 26 metabolites, age, and sex were selected for the first component [shown in A] and 14, six, and five, respectively, and age were selected for the second component. [B] Correlation plots of the first components of the DIABLO analysis using the selected analytes of each analyte type given above, coloour coded as in [A]: green, healthy control; blue, patients in remission; and red, patients with active disease. [C] Illustration of potential pathophysiological mechanisms driving UC disease chronicity as suggested from the present data. Marker molecules detected in tissue samples and in plasma samples are distinguished accordingly and related to the corresponding pathological processes. [D] Z-scores of analytes sorted according their behaviour in the trajectory healthy control [green] -> patients in remission [blue] -> patients with acute disease [red]. Green highlighted are analytes which do not normalise in patients in remission, red highlighted are analytes which get close to normal during remission, and blue highlighted are analytes which show an exceptional behaviour, ie, either the value of healthy controls is in between the other two categories [GUDCA] or the values of healthy controls and patients in remission are at the edges and the values in patients with acute disease are in between. UC, ulcerative colitis.

A second independent analysis approach was solely based on bioinformatical techniques. Analytes-set analyses and data integration analysis were performed with the multiomics data combined with the clinical data, as accomplished previously.^45,71^ This analysis strategy corrected for potential confounders such as age and sex, resulting in updated z-scores of biomarker candidates. Unsupervised hierarchical clustering of the most promising biomarker candidates separated the patient groups, with some intersection between the active colitis and the remission groups [Figure 4A]. When combining eicosanoids with proteins, the resulting PCA fully separated acute patients from the controls, whereas a combination of proteins with metabolites fully separated the remission group from the controls [Figure 4B]. Combining all data separated acute patients from controls, with the remission patients interspersed. A network of correlated significant analytes was generated [Supplementary Figure S3], that may support testing potential disease mechanisms with regard to their data compatibility.

Calculation of z-values, corrected for potential confounders for some biomarker candidates, distinguished three groups of biomarkers [Figure 4C]. Apparently, chronic inflammatory processes and platelet homeostasis failed to normalise upon clinical remission, indicated by SAA1 and ITGA2B, respectively, as average z-values of patients after remission are close to levels of acute UC patients [Figure 4C, marked green]. Other molecules, such as bile acids CA and DCA and the gut microbiome-derived metabolites IndSO_4_, 3-IPA, and 3-IAA, were found close to normal values upon remission, potentially indicating efficient recovery of gut functions such as lipid recovery and gut microbiome metabolism [Figure 4C, marked red]. Remarkably, some chronic inflammation marker molecules, such as SAA4 and PGE2 and the hypoxia marker hypoxanthine, were found even more strongly deregulated in patients after remission when compared with patients suffering from active disease [Figure 4C, marked blue].

The multiomics data were integrated into a disease model composed of distinct pathology-relevant events evidenced by tissue and/or blood-borne biomarkers, as depicted in Figure 4D.

## 4. Discussion

This study presents an in-depth molecular phenotyping of UC pathomechanisms, based on tissue proteomics and multiomics blood plasma analyses. Tissue proteomics confirmed many known local characteristics of UC such as neutrophil invasion and neutrophil extracellular trap as well as scar formation, but also painted a comprehensive picture of acute and chronic pathomechanisms. The plasma profiling data allowed us to assess whether systemic blood-borne molecules might be related to pathological processes detected by tissue proteomics, as outlined in the following.

The need for systems biology and omics datasets to better understand complex diseases such as UC, and the power of the molecular profiling techniques, have been outlined recently. ^35^ Indeed, this study may serve as a typical example for that endeavour, showing potential strengths and opportunities, but also weaknesses and threats. In the following, a potential model for UC pathomechanisms will be presented. We are aware of and will discuss the limitations and potential pitfalls of such an approach.

As expected, many pro-inflammatory mediators were apparently released by tissue-resident immune cells, including neutrophils, macrophages, B cells and T cells [Figure 2; Supplementary data, part 4], inducing inflammatory marker molecules evidenced in blood plasma samples [Figure 3]. Rather unexpectedly, several anti-inflammatory proteins were also found upregulated in inflamed tissue samples. Here we suggest that platelet-derived TGF-β may account for this potentially highly relevant observation, as evidence for UC-associated platelet activation is presented here and platelets are known to release TGF-β upon activation. Thus, the observed upregulation of TNC and AIF1 by macrophages and SERPINB5 and MUC12 by epithelial cells, among others, may be a consequence thereof.

An involvement of platelets in UC pathophysiology was corroborated by tissue proteomics and blood plasma proteomics in addition to blood plasma metabolomics. These data clearly point to the onset of wound-healing activities already during an acute episode of UC [Figure 2C]. However, the present data also suggested regenerative activities contributed by endothelial cells, fibroblasts, and epithelial cells during acute disease states [Figure 2C]. The temporal coincidence of pro- and anti-inflammatory processes represents a hallmark of chronic inflammation, suggesting that unresolved regulatory signalling mechanisms may prevail.^72^ The observed tissue regeneration efforts seem to, at least partially, fail, as the clinical phenotype indicates delayed recovery and a high risk for relapse. This raises the question as to which molecular mechanisms might account for a potential failure of tissue regeneration. The following model may provide an explanation [Figure 4C].

Recent evidence suggested two main contributors to UC initiation: 1] a lack of vagal nerve activities^73^ and a corresponding chronic shortage of blood supply to the gut, resulting in hypoxia and energy deficiency; and 2] an apparent gut microbiome dysbiosis.^74,75^

Mucus formation is an energy-demanding process, and pathogenic microorganisms relevant for UC have been described to degrade mucus.^75^ Thus, these initial processes may result in a loss of mucus, as indicated by some tissue markers. The resulting increased exposure of gut epithelial cells to bacterial endotoxins may eventually trigger inflammation, causing neutrophil invasion and activation. Both tissue and plasma markers may indicate these processes. Hypoxia is capable of directly attracting neutrophils,^76^ and may thus promote the inflammatory response. Exaggerated neutrophil activities may cause tissue and microvascular damage, as indicated by tissue markers, and may further promote local hypoxia.^77^ Epithelial cells will suffer from both neutrophil-induced tissue destruction and a lack of oxygen required for energy-demanding activities, such as mucus synthesis, transcellular transport, and detoxification. A microvascular damage may represent a strong initiator of local platelet activation, indicated by both tissue and blood marker molecules. This evident molecular profile may also relate to the elevated thromboembolic risk of UC patients.^78^ Apparently, a strong angiogenic and wound-healing response is induced by platelets activated at the surface of microvascular damage sites. Activated platelets release TGF-beta,^79^ apparently accounting for a large number of specific protein regulatory events currently observed in inflamed tissue samples. TGF-beta is known to promote myofibroblast and scar formation, processes strongly indicated by tissue marker molecules and known to occur during UC. It is reasonable to expect that scar formation during provisional tissue regeneration results in a further loss of mucus, as fibroblasts are unable to secrete mucus. This would complete a vicious cycle and further promote disease-initiating conditions [Figure 4C].

This disease model may illustrate a major obstacle and challenge for UC treatment. Inhibitors of platelet activation have been comprehensively investigated for UC treatment but apparently failed to deliver a satisfying response.^80^ This suggests that local platelet activation promotes the disease via microvascular closure and the resulting hypoxia and neutrophil activation. On the other hand, platelet activation may be responsible for initiating tissue regeneration by TGF-β release. Remarkably, the present blood plasma proteomics data collected with UC patients after remission strongly suggest that local platelet activation remains to be an ongoing and unresolved disease process. Together with the chronic inflammation marker SAA1, the platelet activation markers ITGA2B, FBLN1, and 11-HETE and the oncometabolite sarcosine apparently remained deregulated. In contrast, the significant downregulation of CRP and several bile acids and microbiome-derived metabolites upon remission suggests successful normalisation of acute inflammation, the gut ,and other gut functions, such as lipid resorption. However, a continuous anti-inflammatory treatment as typically applied for UC patients may also affect stromal cells and thus interfere with regenerative mechanisms in a not yet fully understood fashion.^81^

In general, it seems that a biological prevalence of innate immune system-derived pathogen defence mechanisms above regenerative processes may promote chronicity in UC. Similar observations were reported by us with regard to chemical hepatocarcinogenesis promoted by inadequately activated Kupffer cells,^82^ and a lack of cartilage and tendon regeneration due to pathogen defence activities of neutrophils.^83,84^ In case of UC, the involvement of neutrophils seems to mainly cause tissue destruction, whereas a provisional scar formation may interfere with successful healing by thinning the important mucous layer. These insights may guide novel therapeutic strategies to address the chronicity of UC, supported by the opportunity to directly monitor the corresponding marker molecules in UC patients.

Some limitations of this study need to be outlined. Due to the large number of potential disease players, such as the different cell types apparently involved in UC pathomechanisms, we have refrained from using gene ontology terms for automated analyses. In contrast, we tried to focus on specific events such as marker molecules known to be characteristic for a given cell type and functional state, which may have introduced a somewhat subjective element. Although the causal path analysis software which we used in this study may be best suited to cell culture model systems rather than tissue proteomics, it enabled us to identify the metabolic transcription factor MAX as a potential key factor for understanding the systemic implications of this disease. As this study lacks an inflammatory control comparison cohort such as diverticulitis, we are currently unable to assess the specificity of the observed UC-associated molecular alterations.The large number of observations obtained by multiomics certainly offers the inherent potential to identify possible disease pathomechanisms, but mechanistic studies will be required for their confirmation.

In summary, molecular profiling of tissue and blood plasma samples of ulcerative colitis patients provides insights into pathomechanisms and systemic effects, and identifies novel biomarkers for disease and remission states. The application of a multiomics analysis strategy allowed us to relate systemic blood-borne proteins, metabolites, and lipids to defined UC disease processes evidenced via tissue proteomics. Thus, it was possible to present a potentially prognostic multiomics marker profile which may become useful to assess remission states and responses to therapy in an individualised fashion. Prospective clinical studies are needed to test whether the present biomarker candidates will be able to fulfill our expectation regarding prognostic power supporting disease-modifying treatment strategies for UC.

The proteome analysis datasets presented in this study can be found in online repositories. The mass spectrometry proteomics data have been deposited in the ProteomeXchange Consortium via the PRIDE^39^ partner repository with the dataset identifier PXD030775. Other data underlying this article will be shared on reasonable request to the corresponding author

## Supplementary Material

jjad052_suppl_Supplementary_Data

jjad052_suppl_Supplementary_Table_S1

jjad052_suppl_Supplementary_Table_S2

jjad052_suppl_Supplementary_Table_S3

jjad052_suppl_Supplementary_Table_S4
